# Oxygen Transport through Amorphous Cathode Coatings in Solid-State Batteries

**DOI:** 10.1021/acs.chemmater.3c02351

**Published:** 2024-03-14

**Authors:** Jianli Cheng, Xinxing Peng, Ya-Qian Zhang, Yaosen Tian, Tofunmi Ogunfunmi, Andrew Z. Haddad, Andrew Dopilka, Gerbrand Ceder, Kristin A. Persson, Mary C. Scott

**Affiliations:** † Materials Sciences Division, Lawrence Berkeley National Laboratory, Berkeley, California 94720, United States; ‡ Department of Materials Science and Engineering, University of California at Berkeley, Berkeley, California 94720, United States; § The Molecular Foundry, Lawrence Berkeley National Laboratory, Berkeley, California 94720, United States; ∥ Energy Storage and Distributed Resources Division, Lawrence Berkeley National Laboratory, Berkeley, California 94720, United States

## Abstract

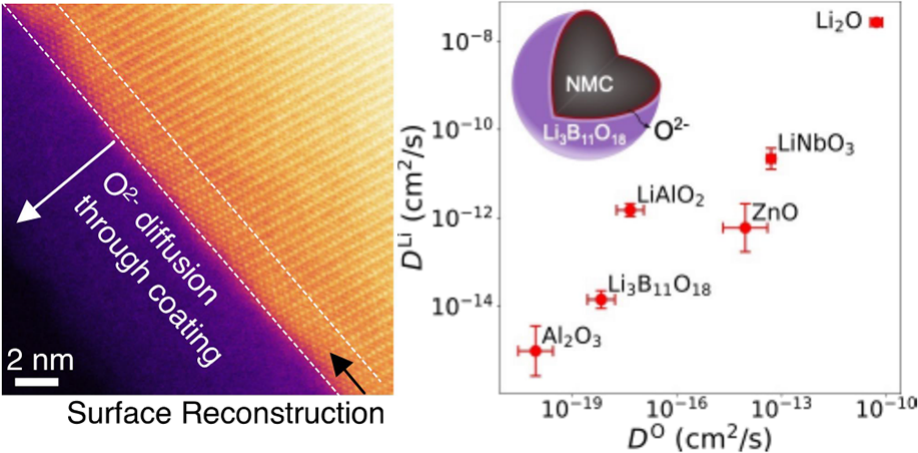

All solid-state batteries (SSBs) are considered the most promising path to enabling higher energy-density portable energy, while concurrently improving safety as compared to current liquid electrolyte solutions. However, the desire for high energy necessitates the choice of high-voltage cathodes, such as nickel-rich layered oxides, where degradation phenomena related to oxygen loss and structural densification at the cathode surface are known to significantly compromise the cycle and thermal stability. In this work, we show, for the first time, that even in an SSB, and when protected by an intact amorphous coating, the LiNi_0.5_Mn_0.3_Co_0.2_O_2_ (NMC^532^) surface transforms from a layered structure into a rocksalt-like structure after electrochemical cycling. The transformation of the surface structure of the Li_3_B_11_O_18_ (LBO)-coated NMC^532^ cathode in a thiophosphate-based solid-state cell is characterized by high-resolution complementary electron microscopy techniques and electron energy loss spectroscopy. Ab initio molecular dynamics corroborate facile transport of O^2–^ in the LBO coating and in other typical coating materials. This work identifies that oxygen loss remains a formidable challenge and barrier to long-cycle life high-energy storage, even in SSBs with durable, amorphous cathode coatings, and directs attention to considering oxygen permeability as an important new design criteria for coating materials.

## Introduction

The rising demand for long-range, safe, electrified transportation has highlighted the need for further advancements in rechargeable Li-ion batteries.^1−4^ Currently, a promising technology to meet these goals is solid-state batteries (SSBs), which provide a safer solution to utilizing higher energy-density electrode materials. Such materials include a Li metal anode and a high-energy density cathode, often found in the family of Ni-rich layered cathodes LiNi_*x*_Mn_*y*_Co_1–*x*_–_*y*_O_2_ (NMC, *x* ≥ 0.5), owing to their high specific capacities and power densities. However, despite the many merits of Ni-rich layered cathodes, numerous studies have shown that when employed in liquid-electrolyte cells, at a high state of charge (SoC), electrochemical oxidation of oxygen and oxygen loss occur in the surface regions of the cathode, leading to destabilization of the transition metal cations at the surface.^5−8^ This destabilization, in turn, leads to surface densification from the initial layered structure (*R*3̅*m*) into an undesirable rocksalt-like (*Fm*3̅*m*) or spinel-like (*Fd*3̅*m*) structure.^7−10^ The resulting structural degradation and interfacial mismatch with the underlying bulk material degrade lithium transport, causing impedance build-up, bulk fatigue,^1^ and poor high-voltage cycling performance.

Indeed, the formation of a structural reconstruction layer (a reduced surface transition-metal layer) has been reported even after simply exposing NMC materials to LiPF_6_-based organic electrolytic solutions.^11^ The most prevalent strategy to protect the cathode surface in traditional liquid electrolyte cells is to coat the cathode with a protective “buffer” layer.^12−14^ This layer typically comprises a solid ceramic^15−20^ and to date, various surface coatings, including metal oxides (e.g., Al_2_O_3_, ZnO, Ta_2_O_5_),^16,17,21,22^ metal fluorides (e.g., AlF_3_),^19,20^ and metal phosphates (e.g., LaPO_4_)^17^ have been shown to enhance the cyclability of NMC materials. However, recently, Zheng et al. reported that although the application of an AlF_3_ coating on Li_1.2_Ni_0.15_Co_0.10_Mn_0.55_O_2_ was found to protect the cathode surface from severe etching/corrosion and alleviated or delayed the surface reconstruction of the cathode compared with that without the coating, phase transformation from the layered to spinel-like structure still occurred after extended cycling.^19^ Similarly, Croy et al.^23^ reported that the use of an atomic layer-deposited Al_2_O_3_ coating as a physical barrier on a Ni-rich cathode was insufficient to overcome its surface instabilities. It is fair to conclude that cathode oxygen loss still occurs in liquid electrolyte cells and that the released oxygen may enhance electrolyte degradation, leading to the formation of high-impedance surface layers. Surprisingly, researchers have recently shown that similar surface structure reconstruction and transition metal reduction can be probed on a bare Ni-rich layered cathode when employed in a thiophosphate-based solid-electrolyte environment.^24^ Ultimately, the final question is whether this surface structure rearrangement can be prohibited with the introduction of a stable, state-of-the-art surface coating^25^ in SSBs.

In this work, we used high-resolution scanning transmission electron microscopy (STEM), transmission electron microscopy (TEM), and electron energy loss spectroscopy (EELS) complemented with ab initio molecular dynamics (AIMD) to investigate the surface structure evolution of a coated Ni-rich layered cathode in a thiophosphate-based SSB environment. An amorphous Li_3_B_11_O_18_ (LBO) coating was selected as an example because, in previous work, it was observed and computed to be chemically/electrochemically stable even after extended cycling in an SSB.^26^ In this work, we show that even when protected by an intact amorphous surface coating, the surface region of LiNi_0.5_Mn_0.3_Co_0.2_O_2_ (NMC^532^) transforms from a layered structure into a rocksalt-like structure after electrochemical cycling in a SSB. More importantly, we demonstrate the essential role of O^2–^ transport in the ceramic coating. To date, surface coatings have been selected based on their facile Li-ion transport, low electron transport, chemical compatibility with both the SE and cathode, and electrochemical stability; however, oxygen transport in the coating material has never been considered as a criterion in their selection. We emphasize that if the surface coating exhibits a facile O^2–^ transport, the cathode can still lose oxygen, densify, and increase its impedance, even when coated by an amorphous surface coating. Using AIMD simulations, we systematically evaluated the O^2–^ diffusion rate in amorphous LBO as well as in other typical coating materials reported in the literature. The O^2–^ flux estimated using the Onsager transport models reveals facile transport of O^2–^ through amorphous LBO, rationalizing our observation that a rocksalt structure still forms on the surface of the NMC^532^ cathode in a solid-state cell. In summary, we provide the first evidence of the formation of a surface-reduced layer in NMC^532^ in the presence of an intact, amorphous oxide coating after cycling in a thiophosphate-based SSB. Our work highlights the importance of designing coating materials with a low O^2–^ diffusivity to mitigate cathode degradation, even in SSBs.

## Methods

### Material Synthesis and Coating Method

The Li_3_B_11_O_18_ (LBO)-coated LiNi_0.5_Mn_0.3_Co_0.2_O_2_ (NMC^532^) samples were prepared by Samsung Research Japan using the sol–gel method reported in previous work.^26^ For the LBO-coated NMC^532^ sample (0.5 mol % LBO coated NMC), the LBO coating solution was first prepared by dissolving a stoichiometric amount of Li acetate and triisopropyl borate in super dehydrated ethanol at 60 °C. Next, the NMC^532^ powder was dispersed in the as-prepared coating solution with stirring. Then, the solvent from the flask was removed using a rotary evaporator in a hot water bath (60 °C) with ultrasound sonication followed by a heat treatment at 350 °C in air. The 75Li_2_S–25P_2_S_5_ (LPS) was synthesized by ball-milling stoichiometric amounts of Li_2_S (99.98% Sigma-Aldrich) and P_2_S_5_ (99% Sigma-Aldrich) in a 50 mL ZrO_2_ jar for 200 min using a SPEX 8000 M mixer mill. The LPS with a small particle size used in the composite cathode was prepared by wet ball milling the LPS solid electrolyte (SE) with heptane and dibutyl ether using a Retsch PM200 ball mill for 40 h. All of the LPS synthesis steps were conducted in an Ar atmosphere.

### Cell Fabrication and Electrochemical Characterization

SSBs were fabricated in an Ar-filled glovebox with H_2_O < 2 ppm and O_2_ < 0.1 ppm. The LPS, uncoated or LBO-coated NMC^532^, and graphite were used as the SE, cathode, and anode active materials, respectively. The cathode and anode composites were prepared by hand mixing 60% active material, 35% LPS, and 5% CNF. The full cells were assembled using an in-house-designed pressure cell (13 mm inner diameter). First, 100 mg of LPS powder was added to the cell and cold pressed under ∼100 MPa pressure. Next, 10 mg of the cathode composite powders and anode composite powders were carefully spread evenly on the top and bottom sides of the LPS pellet, respectively. A pressure of ∼350 MPa was applied for 5 min to compact the cell and ensure good interfacial contact between the different components.^27−29^

All the electrochemical tests were conducted at room temperature under an Ar atmosphere, and a pressure of 5 MPa was applied to the cells during cycling. The full SSBs were cycled at a constant current (0.1 mA cm^–2^ for charge and 0.05 mA cm^–2^ for discharge) between 2.5 and 4.3 V versus Li/Li^+^.

### Electron Microscopy Experiments

The TEM sample preparation was conducted in an Ar-filled glovebox. The LBO-coated cathodes were extracted from the disassembled cells after cycling. The cycled and uncycled composite cathode powder samples were diluted in hexane and sonicated to obtain good particle dispersion. The TEM samples were prepared by drop casting the solution onto a copper mesh TEM grid with an ultrathin carbon and lacey carbon support. The TEM grids were transferred from the glovebox into the microscope under an inert Ar atmosphere using a Gatan 648 double-tilt vacuum-transfer holder. The high-resolution STEM and EELS analyses were performed using the TEAM I microscope (a modified FEI Titan 80–300 microscope with a double-aberration-corrected (scanning) transmission electron microscope) with an accelerating voltage of 300 kV.

For the EELS analysis, a power law background subtraction was performed for the Mn L_3_/L_2_ edge. Then, the multiple scattering was then removed by Fourier-ratio deconvolution using the low-loss spectrum obtained from the same scanning region using the dual EELS mode. To quantify the L_3_/L_2_ intensity ratio, a step function threshold with the ratio of step heights of 2:1 was first applied in the background subtraction to account for the multiplicity of the 3p1/2 and 3p3/2 initial states.^30^ After the step function subtraction, the L_3_/L_2_ intensity ratios were determined by area integration beneath the L_3_ and L_2_ peaks.

### Density Functional Theory Methods

All the density functional theory (DFT) electronic structure calculations were performed using the Vienna Ab initio Simulation Package^31^ with projector-augmented wave potentials.^32^ The Perdew–Burke–Ernzerhof generalized gradient approximation functional was adopted for the exchange–correlation functional.^33^ For the AIMD simulations, we used Γ-point-only Brillouin zone integration at a plane-wave cutoff energy of 400 eV and a time step of 2 fs.

To generate the amorphous structures, we employed a “liquid-quench” process in which heating, equilibration, and quenching were performed using an AIMD workflow, which can be found as part of the open-source mpmorph package at http://github.com/materialsproject/mpmorph. We used the Packmol package^34^ to generate the initial amorphous structures. To generate the “liquid” phase of the amorphous structures, the structures were “heated” at 3000 K, and a sequence of 4 ps AIMD simulations in the *NVT* ensemble was performed until the external pressure and energy converged. Then, the equilibrated “liquid” amorphous structures were simulated for an additional 10 ps, from which three independent configurations were selected, i.e., 3.3 ps apart from each other, and quenched to 0 K to obtain three, representative amorphous structures to exemplify varying atomic environments. The radial distribution functions of B–O, Al–O, and Nb–O pairs in Li_3_B_11_O_18_, LiAlO_2_, and LiNbO_3_, respectively, at 0 K are plotted in Figure S1, which illustrates the distinct ordering behavior between amorphous and crystalline structures. For each configuration, we equilibrated the structures at *T* = 1800, 2000, 2200, 2400, 2600, and 2800 K, respectively, following the same procedure of obtaining the “liquid” amorphous structures mentioned above and then simulated an 80 ps diffusion trajectory at each corresponding temperature. Therefore, from the three representative amorphous structures, there are three independent diffusion trajectories at each corresponding temperature. Further details about the AIMD and DFT workflows can be found in ref.^13^

From the obtained diffusion trajectories, we calculated the self-diffusion coefficients (*D*) of Li^+^ and O^2–^ ions using the Einstein relation: , where *t*, *r*, and  are the time, ion position, and mean square displacement (MSD) that is averaged over all relevant ions (Li^+^ or O^2–^ ions), respectively. Figure S2 illustrates the MSD of the Li^+^ and O^2–^ ions in amorphous Li_3_B_11_O_18_, LiAlO_2_, and LiNbO_3_. From this data, we obtained the *D* values at *T* = 1800, 2000, 2200, 2400, 2600, and 2800 K. At each temperature, there are three *D* values calculated from three independent AIMD diffusion trajectories obtained earlier for each representative amorphous structure. Figure S3 illustrates the density of the amorphous Li_3_B_11_O_18_ structures versus the oxygen diffusivity (*D*^O^). As expected, higher temperatures lead to a lower density due to an expanded volume, which in turn leads to an increase in oxygen diffusivity. The room-temperature *D*_rt_ were extrapolated from the *D* values at high temperatures using the Arrhenius relation, *D* = *D*_0_ exp(−*E*_a_/*k*_B_*T*), where *k*_B_ is the Boltzmann constant, *D*_0_ is the pre-exponential factor, and *E*_a_ is the activation energy of ion diffusion. *D*_0_ and *E*_a_ were determined by fitting log *D* vs 1/*T* as the Arrhenius relation. Compared with the diffusion trajectories at higher temperatures (e.g., 2800 K), the trajectories at lower temperatures (e.g., 1800 K) typically exhibit fewer ion hops, thus yielding fitted *D* values with higher statistical uncertainty. Therefore, we considered the statistical uncertainty of the *D* value at each temperature when fitting the Arrhenius relation by assigning the standard deviation of log *D* as the uncertainty for each averaged *D*. In this study, the Arrhenius relation was fitted by the *curve_fit* function in the SciPy package.^35^ It should be noted that pure B_2_O_3_ glass belongs to a strong glassformer, and its fragility increases with Li_2_O content.^36−38^ Therefore, even though the temperature-dependent *D* in Li_3_B_11_O_18_ follows an Arrhenius-like relation at high temperatures, such as near the glass-transition temperature, it may not follow the same Arrhenius relation at low temperatures, such as room temperature. As a result, our estimated Li^+^ and O^2–^ diffusivities in Li_3_B_11_O_18_ could be overestimated. In addition, we fitted the temperature-dependent diffusivities with a non-Arrhenius Vogel–Fulcher–Tammann (VFT) equation^39^ (see Figure S4), , where *T*_0_ is a temperature that is ∼50 K below the glass-transition temperature. However, because of the time limitations of AIMD simulations in calculating the diffusivities of Li_3_B_11_O_18_ at low temperatures, i.e., 400–800 K, we are not able to obtain the fitted VFT equations with sufficient accuracy.

To estimate the ionic flux under the driving force of the chemical potential gradient across the coating layer, we applied the Onsager transport equation:
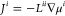
1where *J*^*i*^, *L*^*ii*^, and ∇μ^*i*^ are the flux, Onsager transport coefficient, and chemical potential gradient of species *i*, respectively. It should be noted that in this study, we ignore the contributions from cross-correlations between two distinct species, such as *L*^OLi^, and between distinct sites, such as *L*_distinct_^OO^, to *J*^O^. Assuming steady-state conditions, we can reasonably approximate the chemical potential gradient ∇μ^*i*^ to be a constant throughout the thickness of the coating, which renders the above equation as
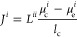
2where μ_c_^*i*^ and μ_e_^*i*^ are the chemical potentials at the cathode and electrolyte sides, respectively. Ignoring the *L*_distinct_^*ii*^ term, *L*^*ii*^ can be directly related to the self-diffusion coefficient *D*^*i*^:
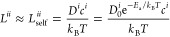
3where *c*^*i*^ is the concentration of species *i* in the coating. The room temperature *L*_rt_^*ii*^ is extrapolated from *L*^*ii*^ values at high temperatures by fitting eq 3.

In this work, we estimate the time *t* required for O^2–^ to diffuse through the LBO coating in order to reach the observed surface rocksalt phase. We assume the surface rocksalt layer of the LBO-coated NMC^532^ mainly consists of NiO phase, which results from the surface NiO_2_ to lose 50% of its oxygen. Let *c*_max_^O^ denote the upper bound value of the O^2–^ concentration in NiO_2_, and *t* can be calculated as
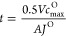
4where *A* and *V* are the surface area of the LBO-coated NMC^532^ cathode particle and the shell volume of the surface rocksalt phase, respectively, and can be expressed as , *A* = 4π(*r* + *l*_c_)^2^, where *r* is the radius of the cathode particle, *l*_s_ is the thickness of the rocksalt phase, and *l*_c_ is the coating thickness. Combining eqs 2 and 4, we obtain
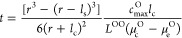
5

As *r* ≫ *l*_s_, *l*_c_, the first term in eq 5 can be considered to be a constant for a given *r*. Therefore, the time *t* required to transport the same amount of O^2–^ through the LBO coating is mainly governed by the values of *l*_c_ and ∇μ^O^. Hence, we consider a range of conditions to estimate *t* by varying the *l*_c_ and ∇μ^O^.

## Results and Discussion

### Appearance of a Surface-Reduced Layer in LBO-Coated NMC upon Cycling

We investigated LBO-coated LiNi_0.5_Mn_0.3_Co_0.2_O_2_ (LBO-NMC^532^) from uncycled and cycled cathode composites (Figures S5–S10). The cathode composites were prepared by hand mixing 60% LBO-NMC, 35% 75Li_2_S–25P_2_S_5_ (LPS), and 5% CNF, with the cycled material undergoing 10 charge–discharge cycles from 2.5 to 4.3 V versus Li. The composite cathode material was then diluted in hexane and sonicated to obtain a good particle dispersion before drop casting on a TEM grid.

**Figure 1:**
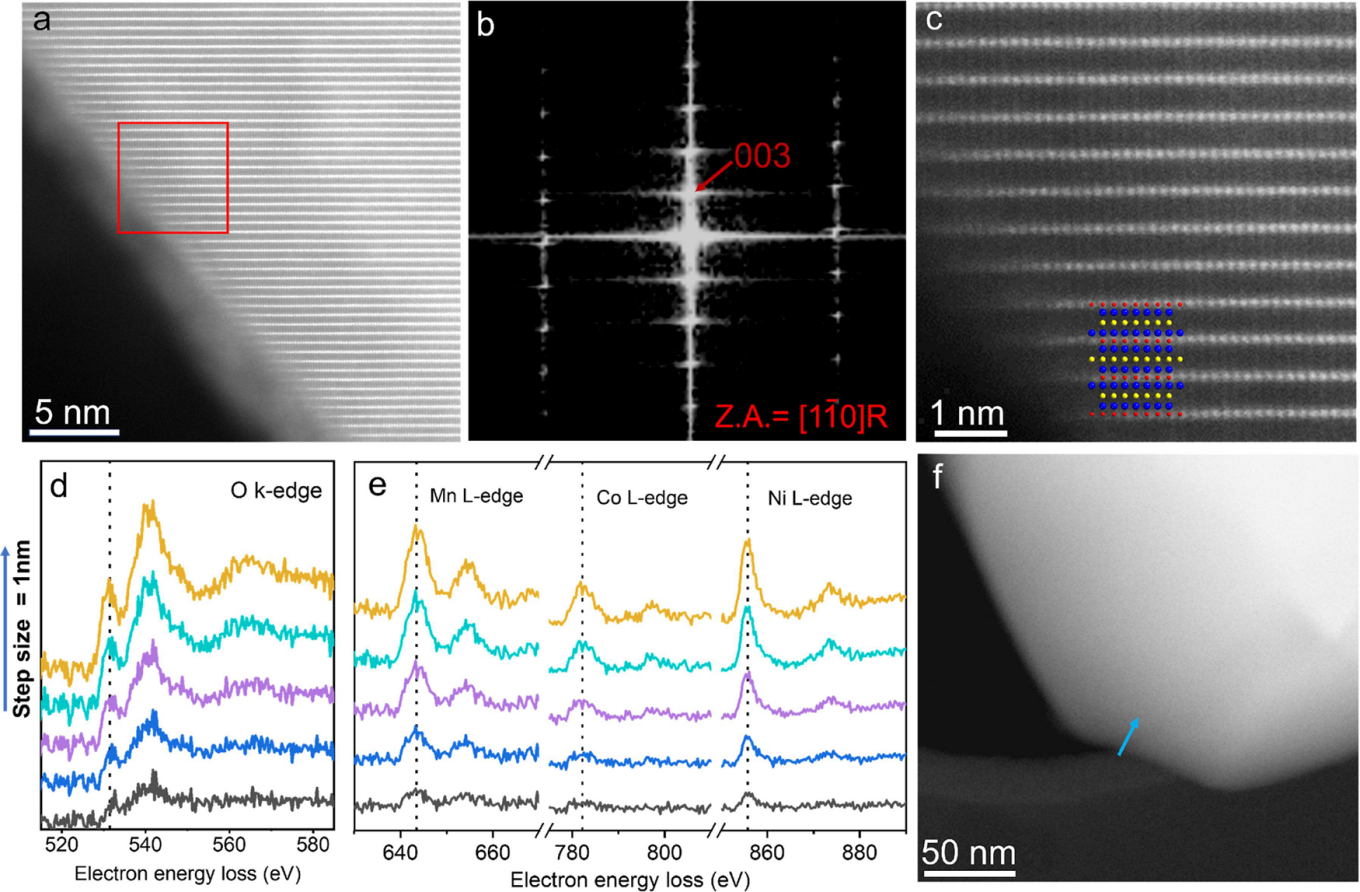
Structural characterization of the uncycled Li_3_B_11_O_18_-coated LiNi_0.5_Mn_0.3_Co_0.2_O_2_ cathode composite. a, HAADF-STEM image of pristine LBO-NMC cathode composite. b, FFT image obtained from the selected surface region, showing the layered rhombohedral *R*3̅*m* structure along the [1–10] zone axis. c, Enlarged HAADF-STEM image of the surface region, confirming the well-defined layered structure prior to cycling. The inset image shows the ball models of NMC^532^ viewing along [1–10]. The red, blue, and yellow balls represent mixed transition metal (Ni, Co, Mn), O and Li atoms. The d, e, EELS line scan spectra of (d) the O–K edge and (e) the Mn, Co, and Ni L-edge collected on pristine LBO-NMC cathode composite along the scanning pathway shown in the HAADF-STEM image in (f).

High-angle annular dark field scanning transmission electron microscopy (HAADF-STEM) images of the uncycled LBO-NMC along the [1–10] zone axis are presented in Figure 1a,c. The bright and dark regions in the HAADF-STEM images correspond to atomic columns of transition-metal cations and Li ions, respectively. Despite coming into contact with the amorphous LPS solid electrolyte (Figure S11), the HAADF-STEM image and corresponding fast-Fourier transform (FFT) image (Figure 1b) indicate that the uncycled nanoscale LBO-coated NMC maintains a well-defined *R*3̅*m* layered structure with separated transition metal and lithium sites in both the edge and bulk of the NMC particle (Figure S9).

Depth-profiled EELS line scan spectra collected on the surface of LBO-NMC from the cathode composite were used to probe the surface and bulk electronic structure of the uncycled LBO-NMC. The oxygen K-edge spectra shown in Figure 1d show a characteristic^40,41^ pre-edge feature onset at ∼525 eV alongside the main edge at 532 eV, which corresponds to the promotion of O 1s electrons to hybridized TM3d-O 2p and TM4sp-O 2p orbitals.^42^ The location and shape of these K-edge features are consistent from the surface to the bulk of the NMC, as are the L edges of the transition metals (Mn, Co, and Ni). These features suggest that the average oxidation states of the transition metal cations remain unchanged in the uncycled LBO-NMC after exposure to the solid-electrolyte thiophosphate environment in the composite cathode.

**Figure 2:**
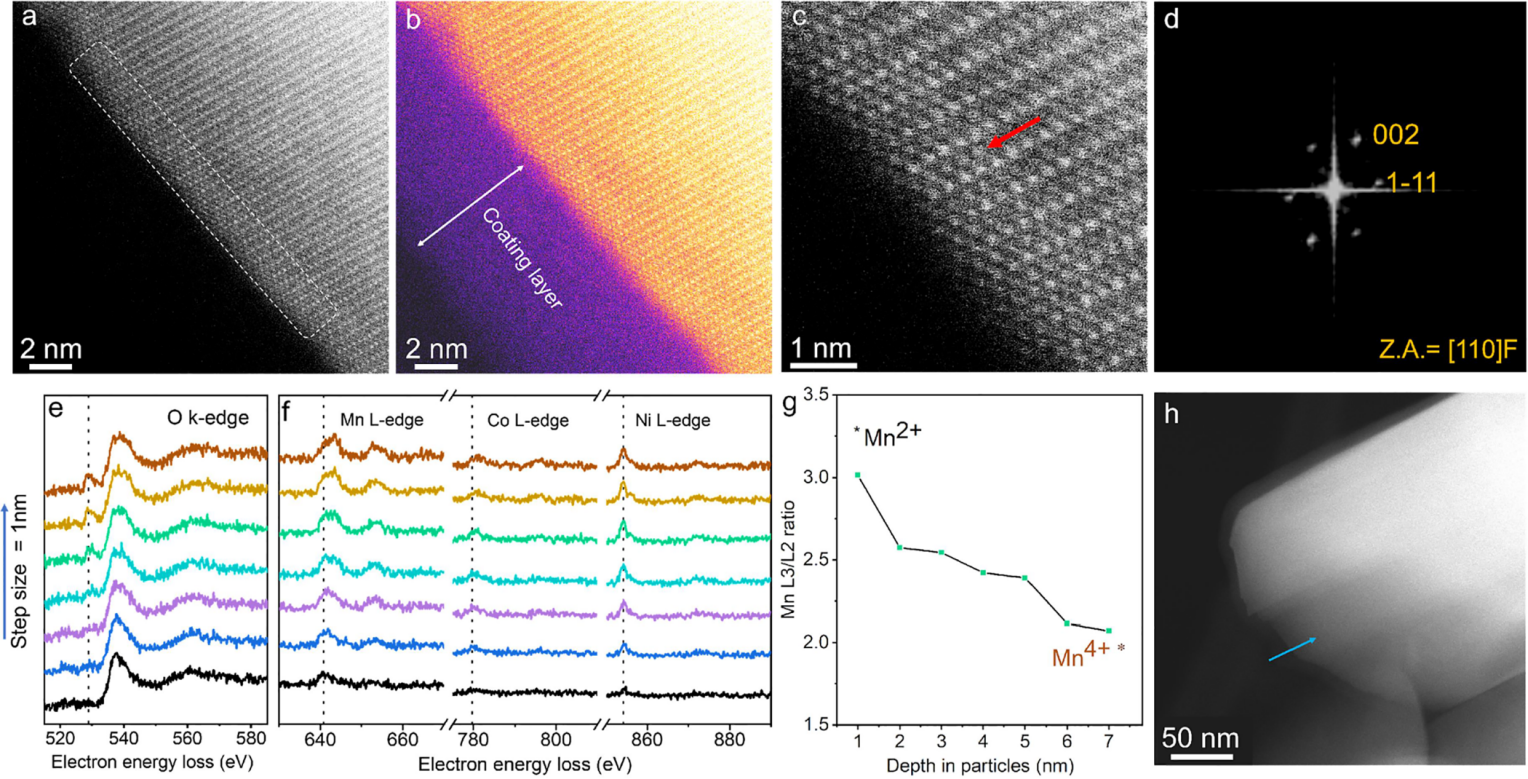
Structural characterization of cycled Li_3_B_11_O_18_-coated LiNi_0.5_Mn_0.3_Co_0.2_O_2_. (a) HAADF-STEM image of LBO-NMC after cycling from 2.5 to 4.3 V versus Li for 10 cycles; (b) the same region displayed with false color for better visualization of the lithium borate thin-film coating. (c) Enlarged HAADF-STEM image of the surface region, showing the surface reconstruction layer. (d) FFT image obtained from the surface region, showing the Fm3̅m rocksalt structure along the [110] zone axis. (e, f) EELS line scan spectra of (e) O–K edge and (f) Mn, Co, and Ni L-edge collected on cycled LBO-NMC along the scanning pathway shown in (h). (g) L_3_/L_2_ intensity ratios of Mn L edges deduced from the EELS spectra in (f). h, HAADF-STEM image, showing the EELS line scan pathway.

A similar analysis of cycled LBO-NMC reveals the presence of a thick rocksalt-like surface layer in the NMC (Figure 2a), despite the nanoscale LBO amorphous coating (Figures 2b, S10, and S12). High-resolution HAADF-STEM imaging (Figure 2a–c) shows the atomic cation arrangement on the surface of the cycled LBO-NMC along the [100] zone axis of the bulk layered rhombohedral *R*3̅*m* structure. Even with the protection of the LBO coating, cation mixing within a few atomic layers (∼2 nm) is readily observed in the surface region of the cycled LBO-NMC (Figures S8 and S10). FFT analysis of the surface of the LBO-NMC after electrochemical cycling confirms that the surface region consists primarily of an *Fm*3̅*m* rock-salt-structured phase (Figure 2d). In addition, the STEM-EELS characterization of the cycled cathode composite confirms a fairly uniform distribution of oxygen in the SE after 10 cycles (Figure S13). Surface reconstruction of the cathode and a change in the oxidation state of the transition metal are observed after cycling (Figures 2a–h, S8, and S10), which supports the coating’s O^2–^ permeability. The presence of oxygen is likely to oxidize Li_3_PS_4_, leading to the formation of a small amount of P–O_*x*_ species.^43^ Further evidence for the formation of a surface-reduced layer on the coated NMC is provided by EELS analysis of the cycled LBO-NMC (Figure 2e–h). As shown in the O K-edge spectra (Figure 2e), the pre-edge of the O K-edge in the surface region shows a significantly reduced intensity compared with that for the uncycled LBO-NMC or the inner region of the cycled NMC particle. Because the pre-edge intensity of the O K-edge strongly correlates with the density of available empty states in the hybridized metal 3d-O 2p orbitals in layered LiTMO_2_,^42^ the area under the oxygen pre-edge is generally found to show a linear correlation with the oxidation state of the TM (Mn, Co, Ni).^44,45^ Thus, the significantly decreased pre-edge intensity on the surface of the cycled LBO-NMC suggests reduced oxidation states of the transition metals in the surface region of the LBO-NMC. The shift toward higher energy of the peak positions in the TM L edge peaks (particularly the Mn and Co L_3_ peaks) at the surface (Figure 2f) also indicates reduced oxidation states near the surface of LBO-NMC after cycling. Quantitative analysis of the Mn L_3_/L_2_ edge intensity ratios in the cycled LBO-NMC shows that in the particle’s bulk, the valence of Mn is 4^+^, but is reduced to ∼2.2^+^ in the surface reconstruction region. The formation of a *Fm*3̅*m* rocksalt structure, the changes in the oxygen pre-edge intensities, and the shift of TM L-edge peaks in the HAADF-STEM and STEM-EELS profiles are all consistent with the presence of a densified surface area comprised of reduced cations on the LBO-coated layered NMC cathode after cycling.^6−8,11^

### Confirmation of Ample Oxygen Mobility in Amorphous Coatings

To validate our hypothesis that oxygen diffusion plays a role in surface densification, we used ab initio methods to evaluate whether sufficient oxygen mobility is present in the coating materials to allow oxygen to escape from the layered cathode surface. We investigated the ionic diffusivity, flux, and transport time through the coating material using AIMD simulations and Onsager transport models. Because of the amorphous nature of the LBO and the typical coatings, we specifically targeted amorphous coatings in our calculations. The equilibrated amorphous structures were generated using a “liquid-quench” process, which was simulated using a series of AIMD calculations, as described in the Methods. From the AIMD-calculated diffusion trajectories, we extracted the self-diffusion coefficients and estimated the O^2–^ diffusion rate.

**Figure 3:**
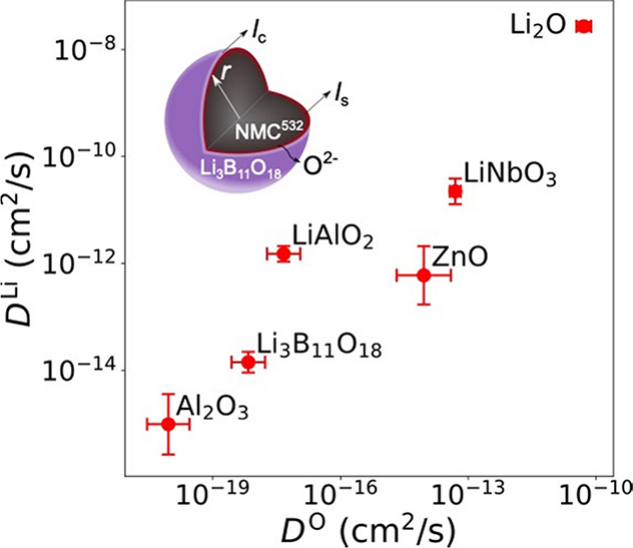
Self-diffusion coefficients in selected amorphous materials. Calculated room-temperature self-diffusion coefficients of Li^+^ and O^2–^ in Li_3_B_11_O_18_, Al_2_O_3_, LiAlO_2_, ZnO, LiNbO_3_, and Li_2_O. The inserted figure shows the oxygen loss from the surface of the Li_3_B_11_O_18_-coated NMC^532^. *r* is the radius of an NMC^532^ particle, *l*_c_ is the coating thickness, and *l*_s_ is the thickness of the surface rocksalt phase. The error bars represent the standard deviation of extrapolated self-diffusion coefficients at 300 K. Amorphous 0.23 Li_2_O·0.77 Al_2_O_3_ and 0.11 Li_2_O·0.89 ZnO were used to simulate Li^+^ and O^2–^diffusion in Al_2_O_3_ and ZnO, respectively.

The room temperature self-diffusion coefficients of Li^+^ (*D*^Li^) and O^2–^ (*D*^O^) in LBO are estimated by extrapolating the *D* values from high temperatures using the Arrhenius relation (Figure S14). For comparison, we also compute *D*^Li^ and *D*^O^ of two commonly used amorphous cathode coatings, LiAlO_2_ and LiNbO_3_, and include three previously calculated^13^ amorphous Al_2_O_3_, ZnO, and Li_2_O, see Figure 3. Figure S14 presents Arrhenius plots of the Li^+^ and O^2–^ self-diffusivity *D* in Li_3_B_11_O_18_, LiAlO_2_, and LiNbO_3_ as a function of temperature *T*. We note that Al_2_O_3_, LiAlO_2_, LiNbO_3_, and ZnO have all been reported to form an amorphous cathode coating and improve the cell cycling stability.^46−48^ The calculated values for *D*^Li^ and *D*^O^ in LBO are 1 × 10^–14^ and 7 × 10^–19^ cm^2^/s, respectively, both of which are lower than the respective values in LiAlO_2_ and LiNbO_3_. Tables 1 and 2 list the calculated activation energies *E*_a_, *D*, and the Onsager transport coefficient *L*^*ii*^ of Li^+^ and O^2–^ diffusion for the amorphous structures. More details on the calculation of *L*^*ii*^ can be found in the Methods. In Figure S15, we also plotted the calculated *D*^Li^ and *D*^O^ at 300 K in various Li_*x*_B_*y*_O_*z*_ compositions among which LBO exhibits the smallest *D*^O^.

**Table 1:** Calculated Activation Energy *E*_a_, Extrapolated Room-Temperature Diffusivity *D*, and Onsager Transport Coefficient *L*^LiLi^ for Li^+^ Diffusion^a^

compounds	*E*_a_^Li^ (eV)	*D*^Li^(cm^2^/s)	error bound *D*^Li^	*L*^LiLi^ (eV^–1^ · cm^–1^ · s^–1^)	error bound *L*^LiLi^
Li_3_B_11_O_18_	0.68 ± 0.03	1 × 10^–14^	9 × 10^–15^, 2 × 10^–14^	6 × 10^9^	4 × 10^9^, 9 × 10^9^
LiAlO_2_	0.54 ± 0.02	2 × 10^–12^	1 × 10^–12^, 2 × 10^–12^	1 × 10^12^	9 × 10^11^, 2 × 10^12^
LiNbO_3_	0.47 ± 0.03	2 × 10^–11^	1 × 10^–11^, 4 × 10^–11^	1 × 10^13^	8 × 10^12^, 2 × 10^13^
Al_2_O_3_	0.74 ± 0.08	1 × 10^–15^	3 × 10^–16^, 4 × 10^–15^	5 × 10^8^	1 × 10^8^, 2 × 10^9^
ZnO	0.58 ± 0.07	6 × 10^–13^	2 × 10^–13^, 2 × 10^–12^	2 × 10^11^	5 × 10^10^, 7 × 10^11^
Li_2_O	0.26 ± 0.01	3 × 10^–8^	2 × 10^–8^, 3 × 10^–8^	7 × 10^16^	6 × 10^16^, 9 × 10^16^

a The error bars correspond to the standard deviation of activation energy from the Arrhenius relation. The error bounds correspond to the standard deviation of extrapolated diffusivities and transport coefficients at 300 K. Amorphous 0.23 Li_2_O·0.77 Al_2_O_3_ and 0.11 Li_2_O·0.89 ZnO were used to simulate Li^+^ diffusion in Al_2_O_3_ and ZnO, respectively

**Table 2:** Calculated Activation Energy *E*_a_, Extrapolated Room-Temperature Diffusivity *D*_rt_, and Onsager Transport Coefficient *L*^OO^ for O^2–^ Diffusion^a^

compounds	*E*_a_^O^ (eV)	*D*^O^ (cm^2^/s)	error bound *D*^O^	*L*^OO^ (eV^–1^ · cm^–1^ · s^–1^)	error bound *L*^OO^
Li_3_B_11_O_18_	0.92 ± 0.05	7 × 10^–19^	3 × 10^–19^, 2 × 10^–18^	1 × 10^6^	5 × 10^5^,4 × 10^6^
LiAlO_2_	0.86 ± 0.05	5 × 10^–18^	2 × 10^–18^, 1 × 10^–17^	9 × 10^6^	3 × 10^6^,2 × 10^7^
LiNbO_3_	0.61 ± 0.02	5 × 10^–14^	4 × 10^–14^, 7 × 10^–14^	9 × 10^10^	7 × 10^10^,1 × 10^11^
Al_2_O_3_	1.04 ± 0.07	9 × 10^–21^	3 × 10^–21^, 3 × 10^–20^	2 × 10^4^	5 × 10^3^,6 × 10^4^
ZnO	0.67 ± 0.09	9 × 10^–15^	2 × 10^–15^, 4 × 10^–14^	1 × 10^10^	3 × 10^9^,6 × 10^10^
Li_2_O	0.37 ± 0.02	5 × 10^–11^	3 × 10^–11^, 8 × 10^–11^	7 × 10^13^	5 × 10^13^,1 × 10^14^

a The error bars correspond to the standard deviation of the activation energy from the Arrhenius relation. The error bounds correspond to the standard deviation of extrapolated diffusivities and transport coefficients at 300 K. Amorphous 0.23 Li_2_O·0.77 Al_2_O_3_ and 0.11 Li_2_O·0.89 ZnO were used to simulate O^2–^ diffusion in Al_2_O_3_ and ZnO, respectively.

We evaluate the effectiveness of the LBO coating in suppressing oxygen loss-induced surface reconstructions of the layered cathode by estimating the O^2–^ flux under steady-state conditions. To estimate the time *t* required for O^2–^ to diffuse through the LBO coating, we consider a range of conditions by varying the coating thickness (*l*_c_) and oxygen chemical potential gradient (∇μ^O^). The oxygen chemical potential on the cathode side (μ_c_^O^) can be estimated from the cathode densification reaction consistent with the phase diagram. For example, at 4.3 V, layered NiO_2_ would densify to rocksalt NiO, with oxygen being released at μ_c_^O^ = −4.95 eV. The estimate for the oxygen chemical potential on the electrolyte side (μ_e_^O^) depends on how the oxygen reacts on the electrolyte side of the coating. We evaluate two limiting conditions: (1) a lower bound for the chemical potential obtained from reaction of oxygen with the electrolyte to form a new oxidized compound (at μ_e_^O^ = −8.39 eV, Li_3_PS_4_ reacts with oxygen and forms Li_3_PO_4_); (2) an upper bound assuming oxygen loses electrons to the carbon network and is released as O_2_ (at μ_e_^O^=–5.24 eV at room temperature and *P*_e_^O_2_^ = 0.21 atm). Therefore, the range of μ_e_^O^ is assumed to be −8.39 ≤ μ_e_^O^ ≤ −5.24 eV. The O^2–^ concentration in NiO_2_, denoted as *c*_max_^O^, is ≈4.2 × 10^22^ cm^–3^. Thus, densification to NiO requires about 2.1 × 10^22^ cm^–3^ of O^2–^ to diffuse through the coating. Based on the experimental observations, we set the radius of the NMC^532^ cathode particle to *r* = 5 μm and the thickness of the surface rocksalt phase to be *l*_s_ = 2 nm. Finally, we estimated the time *t* required for the surface NiO_2_ to lose 50% of its oxygen. Table 3 shows that the released oxygen can diffuse through the LBO coating in 1.4–165 min, depending on *l*_c_ and ∇μ^O^. We emphasize that this time is considerably shorter than the time an electrode spends at high voltage, where its driving force for oxygen release is the highest.

**Table 3:** Estimated Time *t* for O^2–^ to Diffuse through the LBO Coating for Various *l*_c_ Values and ∇μ^*i*^^a^

*r* (μm)	*l*_s_ (nm)	μ_c_^O^ (eV)	μ_e_^O^ (eV)	*l*_c_ (nm)	*t* (min)	error bound *t*
5	2	–4.95	–8.39	1	1.4	0.5, 3.8
				10	14	5, 38
			–5.24	1	16.6	6.5, 45
				10	165	64,452

a *l*_c_ is the LBO coating thickness. μ_c_^O^ and μ_e_^O^ are the oxygen chemical potentials on the cathode and electrolyte side, respectively. We assume an NMC^532^ cathode particle radius (*r*) of 5 μm that forms a surface layer of densified NiO rocksalt phase with thickness (*l*_s_) of 2 nm.

### Implications for Cathode Coating Design

In this article, we provide the first evidence of the formation of a surface-reduced layer in NMC^532^ in the presence of an amorphous LBO coating after cycling in a thiophosphate-based SSB. The fact that a reduced cation-disordered region forms at the surface of LBO-NMC^532^ indicates that the strategy of using a chemically/electrochemically stable amorphous surface coating with low electronic conductivity remains insufficient to inhibit the surface densification of the cathode particles, even in a SSB. We propose that the origin of the surface transformation of NMC^532^ in the presence of an amorphous coating originates in oxygen transport through the coating. Using AIMD and the Onsager transport model, we demonstrate that under a reasonable gradient range of oxygen chemical potential and coating thickness, transport of the O^2–^ through the LBO coating can indeed occur on the time scale of electrochemical cycling. Such oxygen loss triggers the formation of a densified rocksalt phase at the surface of NMC^532^, even in the presence of an intact and amorphous surface coating. Besides increasing the impedance of the cathode, oxygen transport through the coating may damage the solid electrolyte by creating phases with lower conductivity or by mechanical decohesion related to the reaction-induced volume change.

As revealed by the modeling, the effectiveness of a surface coating in retaining oxygen in the cathode is determined by both kinetic and thermodynamic factors, primarily the oxygen diffusion rate in the coating materials and the oxygen chemical potential gradient across the coating layer. The high oxygen chemical potential of a charged cathode drives the O^2–^ to diffuse from the cathode surface to the SE. On the cathode surface side, O^2–^ can either lose electrons to the carbon network and be released as O_2_ or react with the SE. In an SSB with a thiophosphate SE, the presence of oxygen is likely to oxidize Li_3_PS_4_.^49,50^ Although previous research has shown that for an uncoated Ni-rich layered cathode, the surface oxygen loss and surface structure rearrangement is strongly correlated to the environmental conditions,^51^ our calculations confirm that it is possible to create a surface reduced layer even when direct contact between the cathode and the SE is prevented by an amorphous coating. It is worth noting that although the amorphous LBO coating significantly improves the cycling performance of full SSBs compared with an uncoated NMC cathode (Figures S16 and S17), a capacity loss of ∼20% is still observed after 100 cycles. The reduced Li kinetics due to cathode surface densification and the oxidation of the surrounding thiophosphate SE all play roles in this performance degradation.

Although our key findings in this study are based on a thiophosphate system, we argue that oxygen diffusion in the coating material is broadly relevant in solid-electrolyte and liquid-electrolyte systems. For a solid electrolyte that does not react with oxygen, such as Li_7_La_3_Zr_2_O_12_, μ_e_^O^ can be estimated from the oxygen partial pressure (*P*_e_^O_2_^) of air at room temperature and therefore corresponds to the calculated upper bound of μ_e_^O^ = −5.24 eV. In this case, we find that the driving force for oxygen diffusion through the coating becomes smaller than in the case of a reactive SE such as Li_3_PS_4_. However, in this scenario, we still estimate that only 17–165 min is needed at a high state of charge for enough oxygen to diffuse through a 1–10 nm LBO coating to create a 2 nm fully densified layer. This diffusion time is comparable to the thiophosphate case and, in the context of SSB operation, is expected to occur within a few cycles.

To demonstrate the broader consequences of our findings, we extend our modeling to other reported coating materials. Following the same procedure and assumption outlined above, we determine O^2–^ transport through a 1–10 nm Al_2_O_3_ coating. We find that the O^2–^ diffusivity (*D*^O^) and Onsager transport coefficient (*L*^OO^) in Al_2_O_3_ are approximately 2 orders of magnitude less than those in an LBO coating (see Table 2). As a result, the estimated time *t* required to transport the same amount of O^2–^ through an Al_2_O_3_ coating varies between 2 and 230 h, depending on *l*_c_ and ∇μ^O^ (see Table S1). Experimentally, Croy et al.^23^ reported that Al_2_O_3_ surface coating is not sufficient to stabilize an NMC cathode surface, while David et al.^16^ showed an Al_2_O_3_ ALD coating can effectively prevent surface reconstruction of an NMC cathode. Our calculations demonstrate that an NMC cathode coated with a thin Al_2_O_3_ coating layer, such as 1 nm, is still prone to surface oxygen loss, especially when cycled at a low C-rate. On the other hand, a thicker Al_2_O_3_ coating layer can effectively mitigate O^2–^ transport, which results in better cathode surface protection. In addition, our calculations show that the Li^+^ and O^2–^ diffusivities in commonly used LiAlO_2_ and LiNbO_3_ coatings are higher than those of LBO, which suggests a faster oxygen loss should occur from LiAlO_2_- or LiNbO_3_-coated NMC. Other inorganic Li^+^-containing compounds, such as Li_2_CO_3_, have been suggested as viable components in a protective surface coating layer due to their electronic insulating properties and overall acceptable ionic conductivity. A previous theoretical study has reported that the Li^+^ diffusivity in Li_2_CO_3_ is ∼10^–7^ cm^2^/s.^52^ Given the correlated diffusion between Li^+^ and O^2–^, we approximate that Li_2_CO_3_ exhibits a similar O^2–^ diffusivity with Li_2_O, ∼10^–10^ cm^2^/s. It should be noted that our diffusion analysis neglects the reaction kinetics of surface structural transitions and back diffusion of transition metals whose effects contribute to the surface reconstruction into the densified layer.^9^ The bulk region of the cathode particle remains as a layered phase due to the slow kinetics associated with the layered-to-spinel transition.^9^

Using ab initio calculations and the Onsager transport theory, we propose that oxygen transport in coating materials plays an essential role in the surface reconstruction and oxygen loss of the layered cathode in various coating and electrolyte systems. In addition to the criteria of providing facile Li^+^ transport and preventing chemical reactions between the cathode and electrolyte, we highlight the importance of designing coating materials with low O^2–^ diffusivity to block oxygen diffusion and mitigate lattice densification at the cathode surface. However, based on the coating materials investigated in this study, we note that there can be a trade-off between the O^2–^ diffusivity and Li^+^ diffusivity, as shown in Figure 3. Generally, the diffusion of Li^+^ and O^2–^ are correlated.^14^ This is because Li^+^ is bonded to its neighboring O^2–^ ions, and its diffusion through the amorphous coating is governed by discrete hops between two adjacent sites, which are initiated by the Li–O bond breaking/formation process. Therefore, more sluggish O^2–^ diffusion generally accompanies a slower Li^+^ diffusion. An ideal amorphous coating should maintain a low O^2–^ diffusivity and a high Li^+^ diffusivity to achieve oxygen-retaining and surface-protective functions while avoiding significant losses in rate capacity. To guide the selection of coatings with adequate Li^+^ diffusion as well as reasonably low O^2–^ diffusion, we refer to ref^14^ which presents an extensive high-throughput computational study of 20 common coating materials and their self-diffusion coefficients.^14^ In ref,^14^ design guidelines for Li^+^ and O^2–^ diffusivities are provided; specifically recommending that Li^+^ diffusivity should be higher than 7 × 10^–16^ cm^2^/s and O^2–^ diffusivity should be lower than 10^–17^ cm^2^/s. In particular, it is reported that BO_*x*_^*y*–^, SiO_*x*_^*y*–^, PO_*x*_^*y–*^, and SbO_*x*_^*y–*^ exhibit improved oxygen retention,^14^ which focuses the attention on cathode coating materials that contain one or more of these anion groups.

## Conclusions

In this work, we use STEM and EELS to conclusively show that even in an SSB, and when protected by an intact, amorphous coating, the surface of a high-energy density oxide cathode still transforms from a layered structure into a rock-salt-like structure after electrochemical cycling. We propose that the reason a stable surface coating cannot inhibit the cathode surface degradation lies in oxygen transport in the surface coating. Using AIMD calculations, we systematically evaluated the O^2–^ diffusion rate in amorphous LBO as well as in typical cathode coating materials reported in the literature. Our results demonstrate the facile O^2–^ transport through the LBO coating and explain similar surface densification observed in liquid-cell systems on coated layered oxide cathodes in the literature. This work identifies oxygen loss as a significant barrier to long-cycle life high-energy storage, even in SSBs with coated cathodes, and highlights the need to design durable, amorphous cathode coatings with optimized lithium/oxygen diffusivity.

## Data Availability

All data needed to evaluate the conclusions in the paper are present in the paper and/or the Supporting Information.

## Supporting Information Available

Radial distribution functions, MSD of Li^+^ and O^2–^ ions, density versus oxygen diffusivity, fitting diffusivities using VFT and Arrhenius equations, TEM images of the uncycled and cycled cathode, structural characterization of the uncycled and cycled cathode, X-ray diffraction pattern of LPS, thickness distribution of the LBO coating layer, composition characterization of the cycled cathode, Arrhenius plots of Li^+^ and O^2–^ diffusivity, calculated room-temperature diffusivities of Li^+^ and O^2–^, cycling performance comparison, electrochemical cycling data, and estimated time for O^2–^ to diffuse through the Al_2_O_3_ coating (PDF)

## Accession Codes

The codes used in this study are available either publicly (mpmorph; http://github.com/materialsproject/mpmorph) or through subscription (Vienna Ab-initio Simulation Package; https://www.vasp.at). For a detailed description of input parameters used for each code, refer to the “Density functional theory methods” section in the Methods.
