# Supplementary material for: Oxygen Transport through Amorphous Cathode Coatings in Solid-State Batteries
Source: Chem Mater. 2024 Mar 14;36(6):2642–51. doi: 10.1021/acs.chemmater.3c02351 (PMC10976630; doi:10.1021/acs.chemmater.3c02351)
Supplement: Supplementary file 1 — cm3c02351_si_001.pdf [file cm3c02351_si_001.pdf]

Oxygen Transport through Amorphous Cathode Coatings in Solid-State Batteries

Jianli Cheng [a†], Xinxing Peng [b,c†], Ya-Qian Zhang [b,c†], Yaosen Tian [a,b], Tofunmi Ogunfunmi [b], Andrew Z. Haddad [d], Andrew Dopilka [d], Gerbrand Ceder [a,b*], Kristin A. Persson [a,b,c*], Mary C. Scott [b,c*]

a. Materials Sciences Division, Lawrence Berkeley National Laboratory, Berkeley, CA 94720, USA

b. Department of Materials Science and Engineering, University of California at Berkeley, Berkeley, CA 94720, USA

c. The Molecular Foundry, Lawrence Berkeley National Laboratory, Berkeley, CA 94720, USA

d. Energy Storage and Distributed Resources Division, Lawrence Berkeley National Laboratory, Berkeley, CA 94720, USA

†Co-authorship

[*]Corresponding author

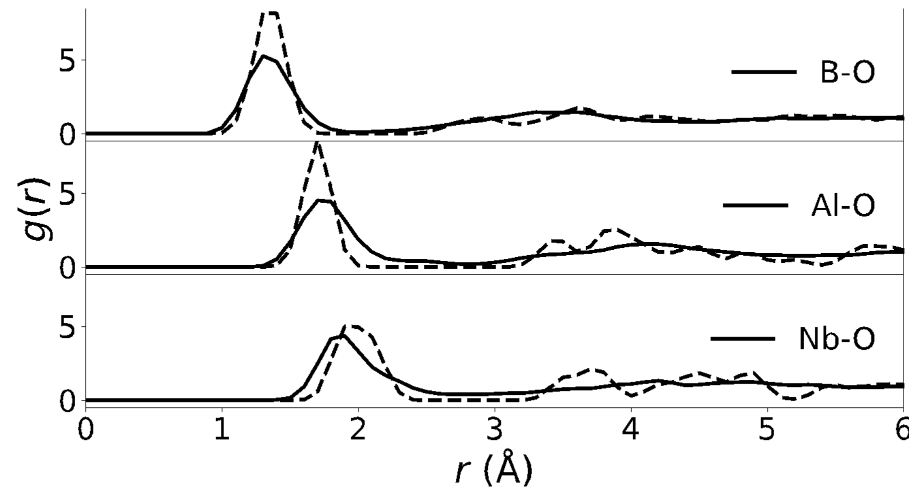

Figure S1. Radial distribution functions of B–O, Al–O, and Nb–O pairs in $Li_3B_{11}O_{18}$, $LiAlO_2$, and $LiNbO_3$, respectively. The dashed curves represent crystalline structures, and the solid curves represent amorphous structures.

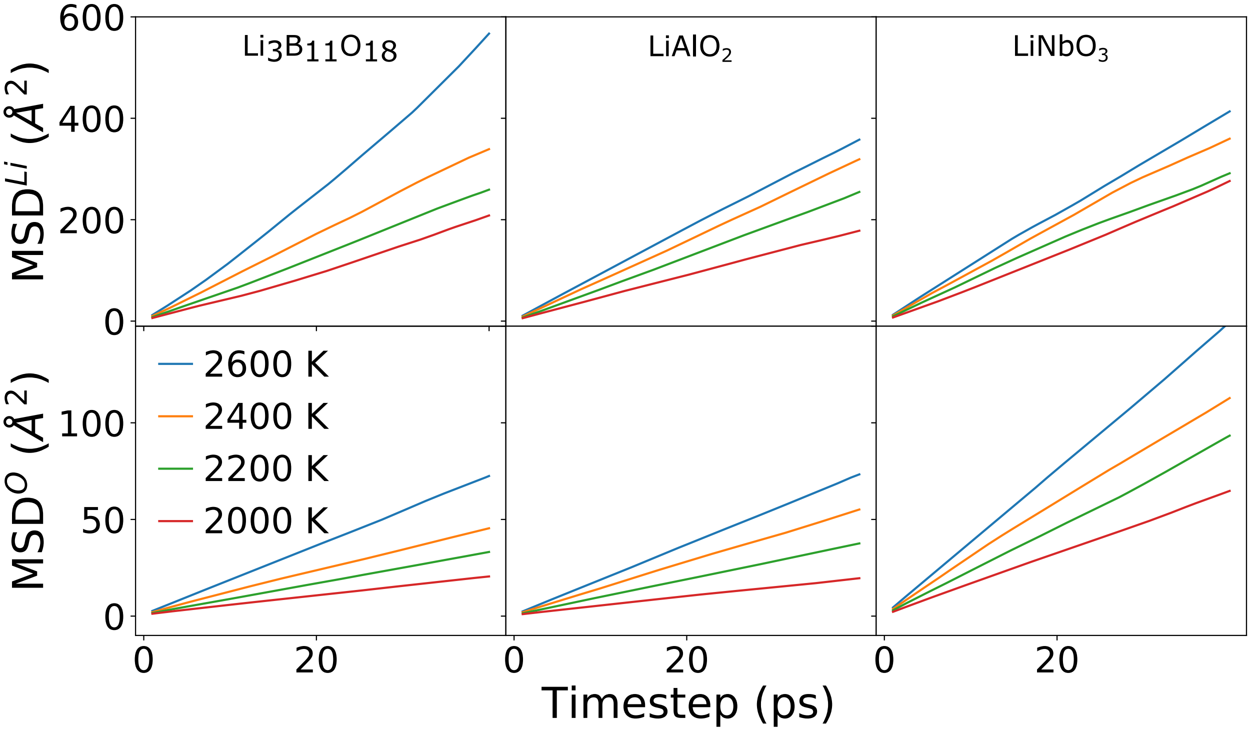

Figure S2. MSD of Li^{+} and O^{2-} ions as a function of timestep in amorphous $Li_3B_{11}O_{18}$, $LiAlO_2$, and $LiNbO_3$. Each MSD vs time step plot was generated using 200 data points, with each data point 0.2 ps apart and the largest timestep is 40 ps.

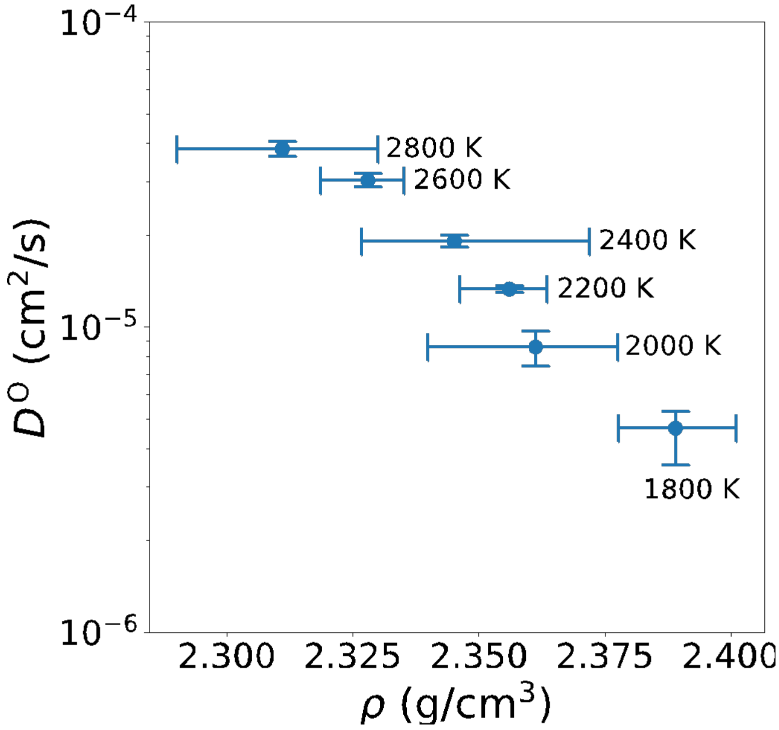

Figure S3. Density of the amorphous $Li_3B_{11}O_{18}$ structures versus the oxygen diffusivity. The error bars represent the spread of the calculated density (ρ) and oxygen diffusivity (D^{O}).

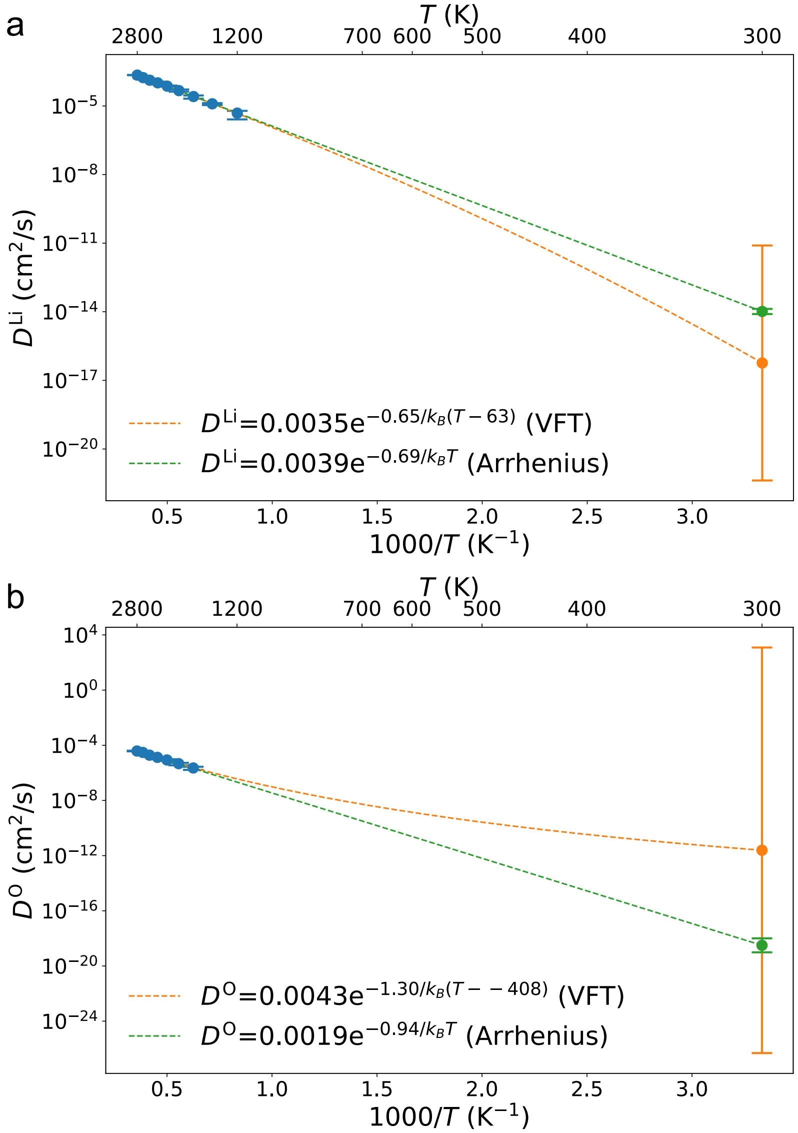

Figure S4. Comparison of the fitted temperature-dependent diffusivities using VFT and Arrhenius equations for (a) Li^+ and (b) O^{2-} diffusions in $Li_3B_{11}O_{18}$.

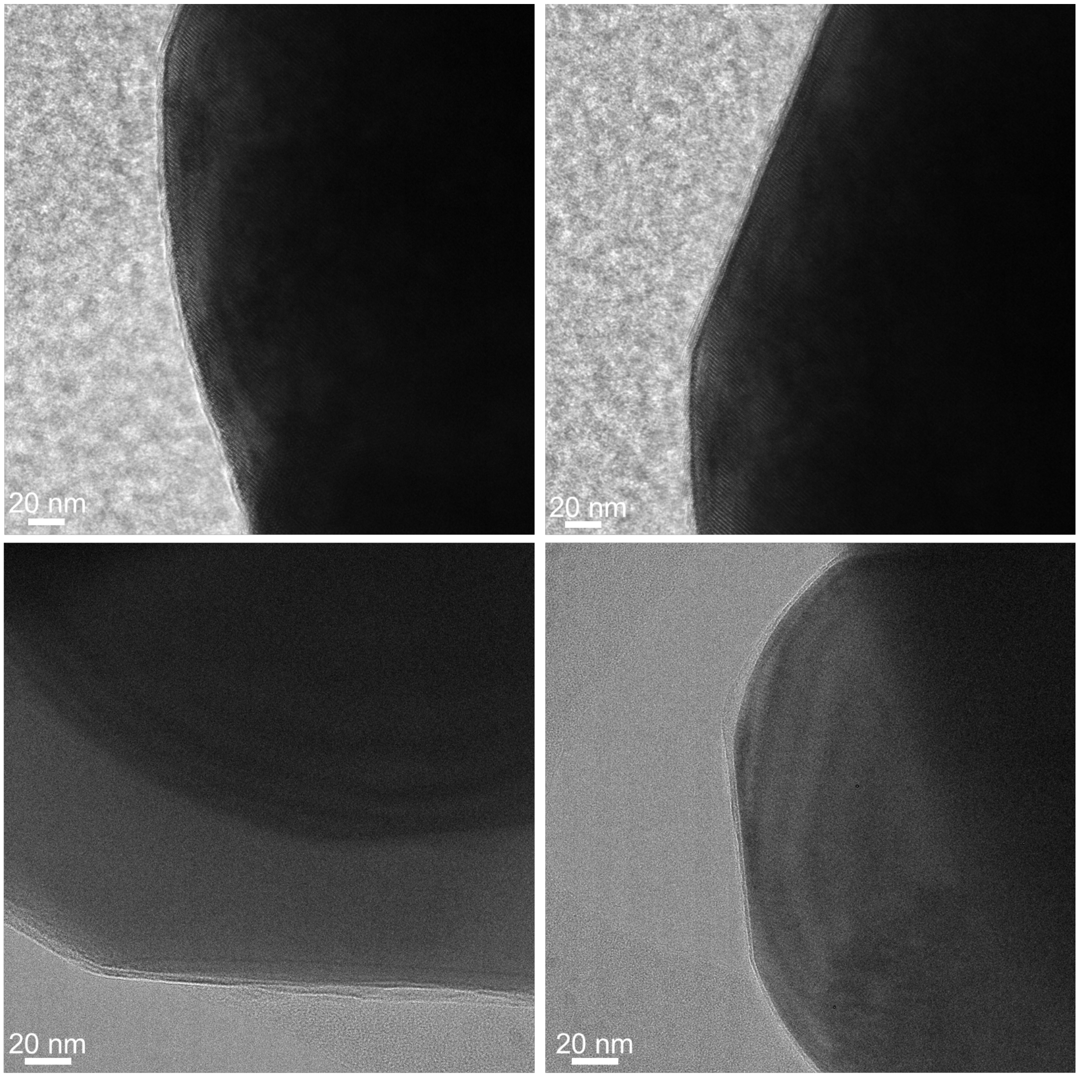

Figure S5. Low-magnification TEM images of uncycled LBO-coated NMC^{532} cathode. A uniform coated layer was observed on the surface of NMC^{532} particles.

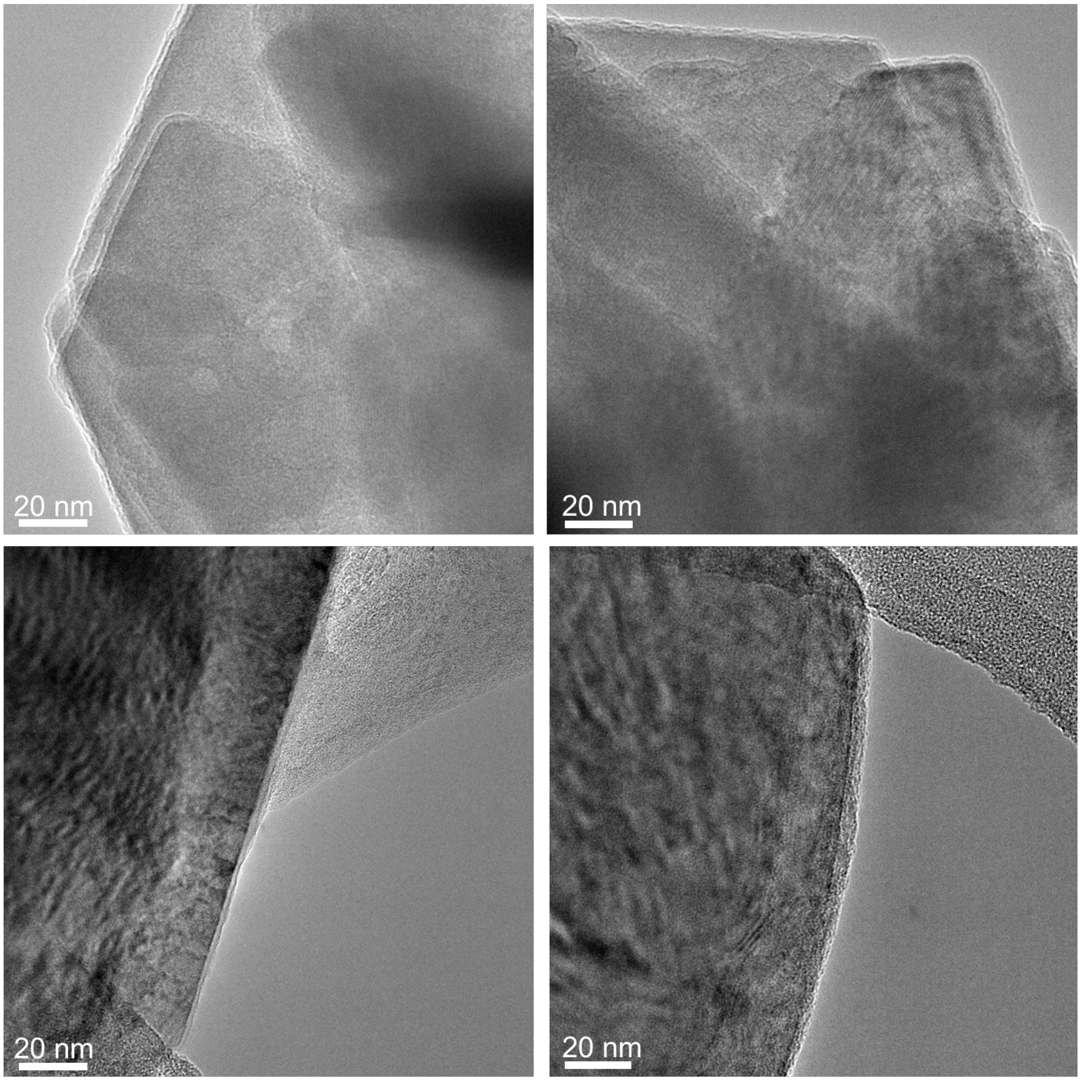

Figure S6. Low-magnification TEM images of cycled LBO-coated NMC[532] cathode. The coating layer is still observed after 10 cycles.

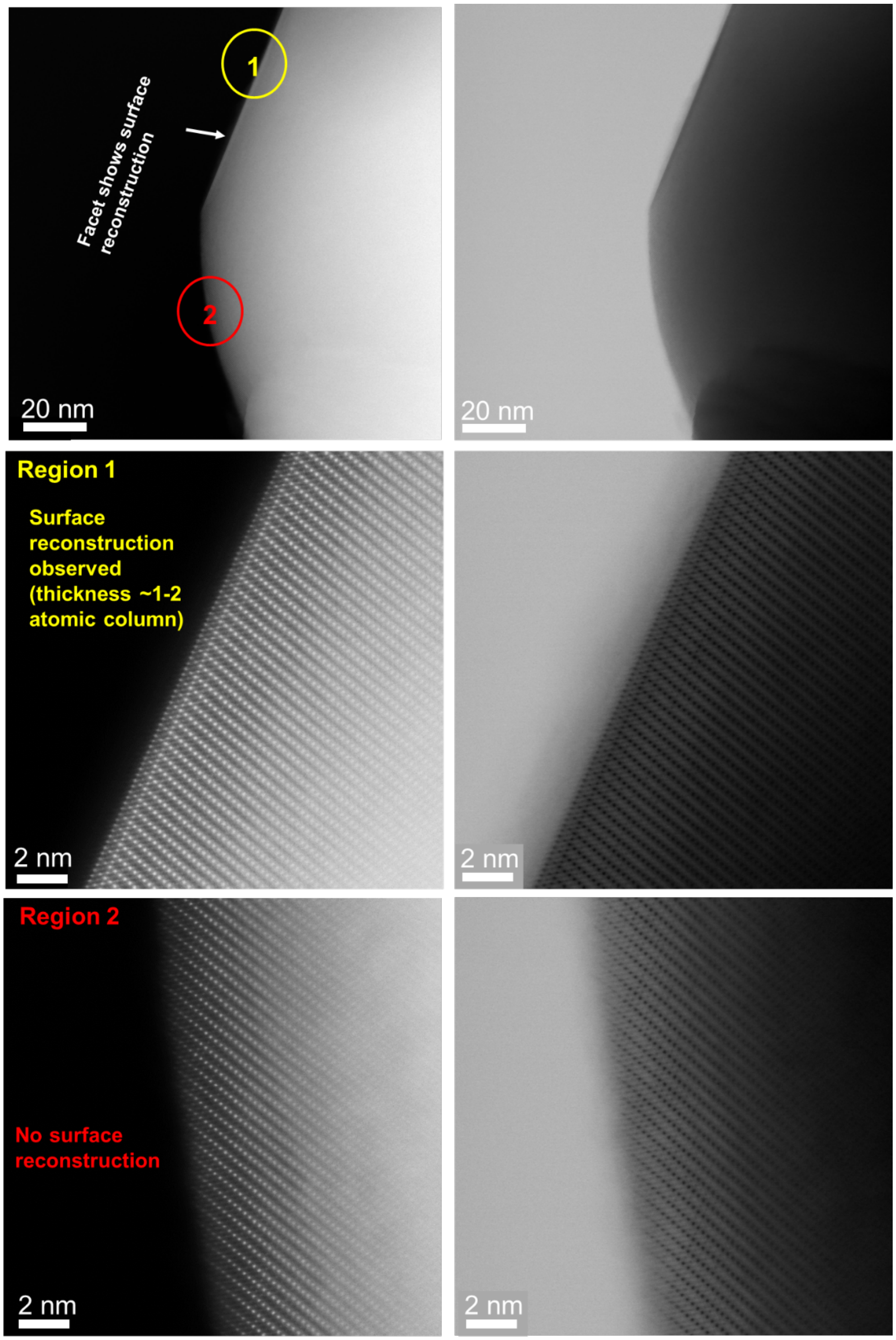

Figure S7. Structural characterization of uncycled LBO-NMC^{532} cathode material. HAADF-STEM (left panel) and bright field (BF) STEM (right panel) images of pristine LBO-NMC^{532} particles.

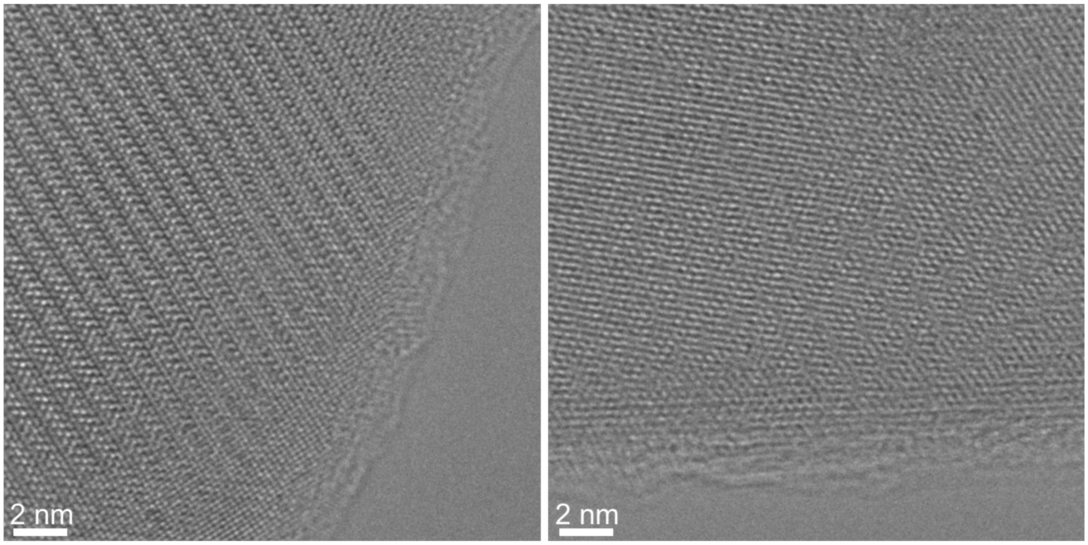

Figure S8. Structural characterization of cycled LBO-NMC[532] cathode material. High-resolution TEM images of LBO-NMC[532] particles after 10 cycles.

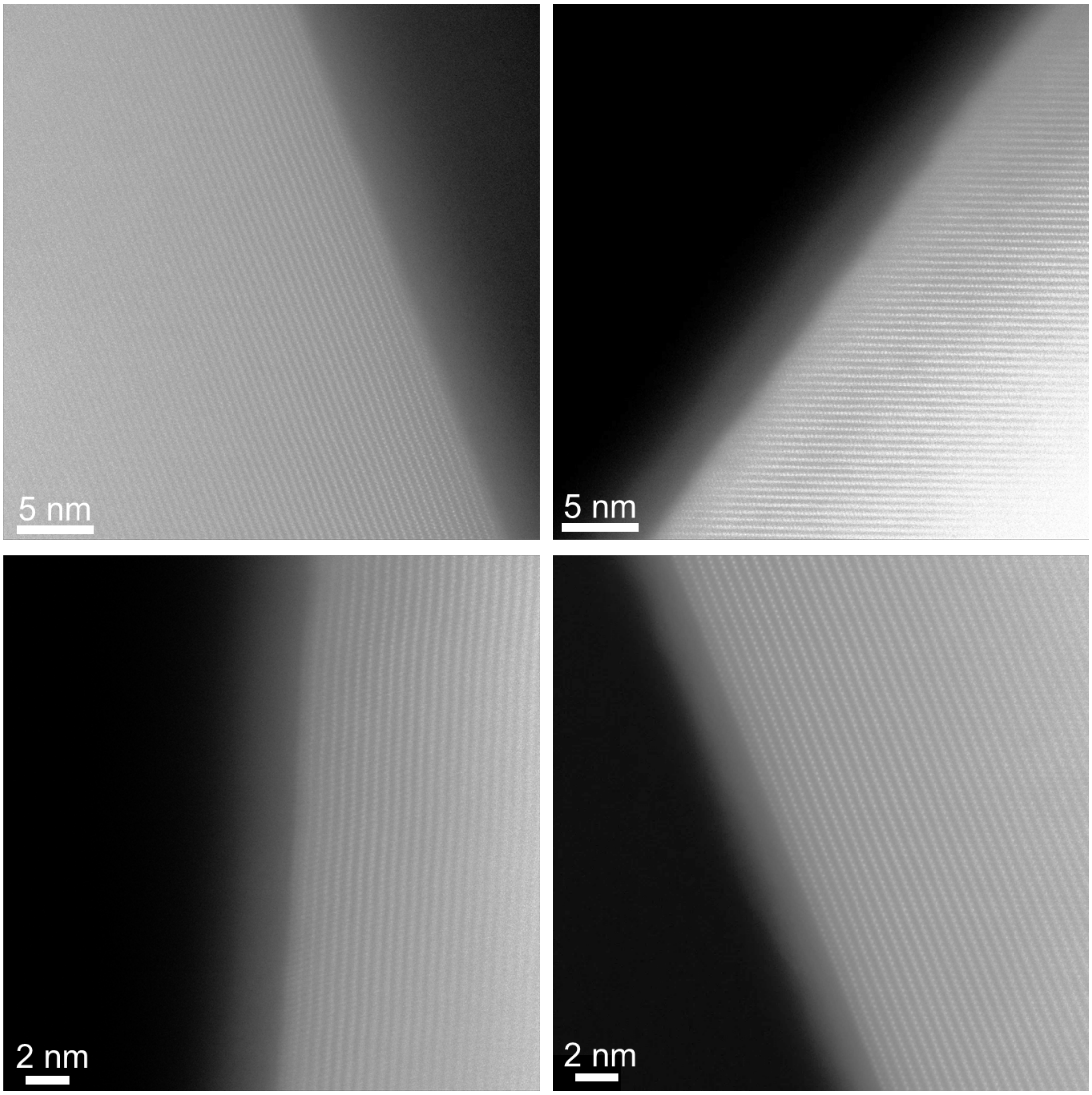

Figure S9. Structural characterization of uncycled LBO-NMC[532] cathode material. High-resolution STEM images of pristine LBO-NMC[532] particles.

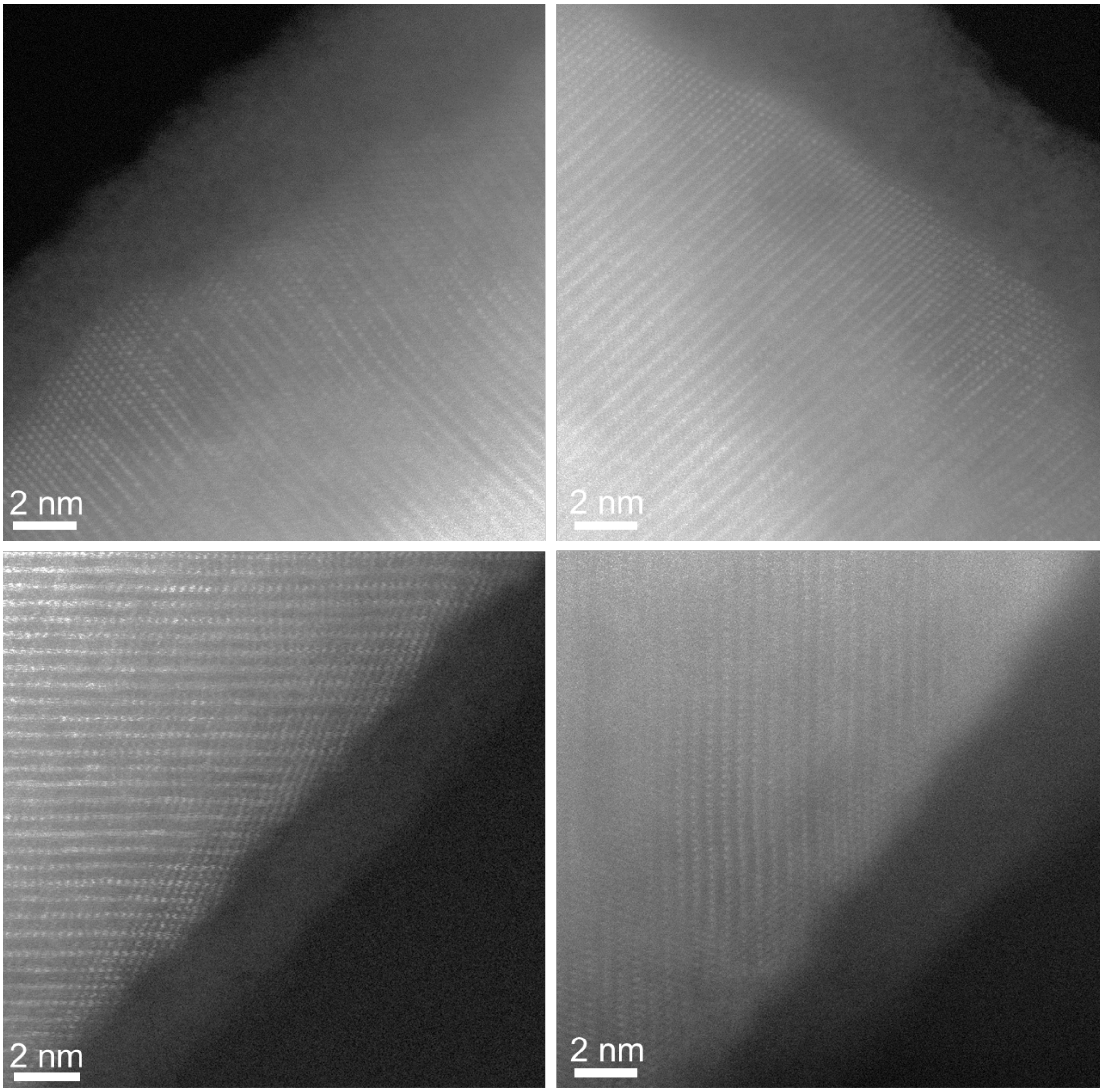

Figure S10. Structural characterization of cycled LBO-NMC^{532} cathode material. High-resolution STEM images of LBO-NMC^{532} particles after 10 cycles.

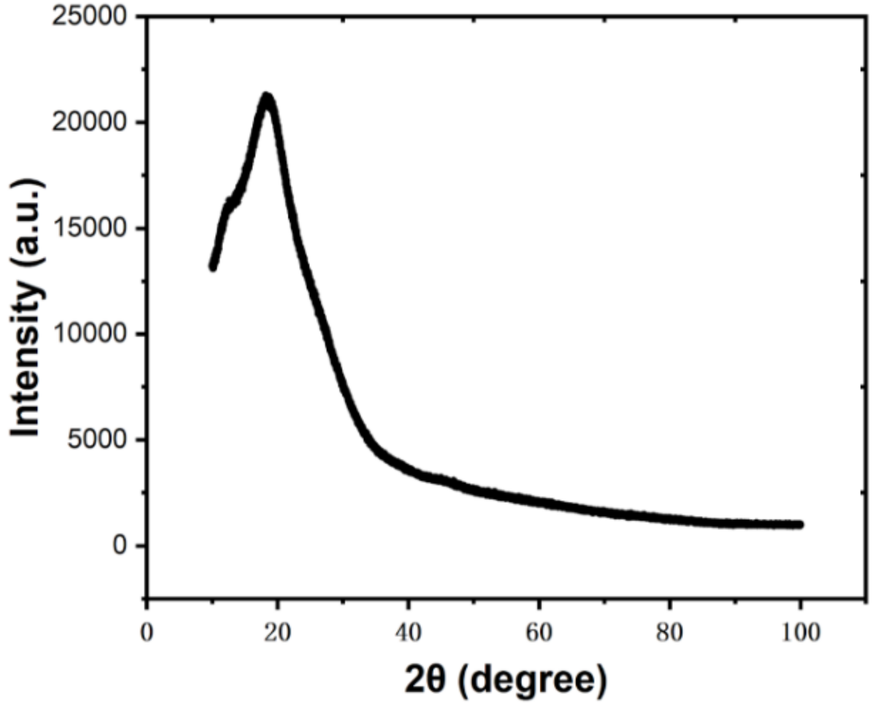

Figure S11. X-ray diffraction pattern of synthesized LPS materials

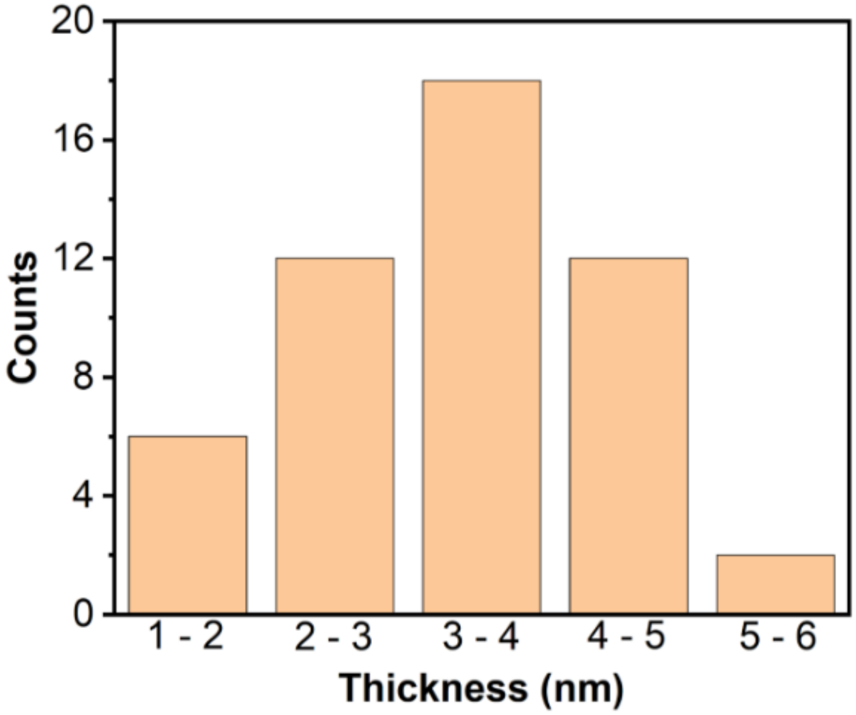

Figure S12. Thickness distribution of the LBO coating layer

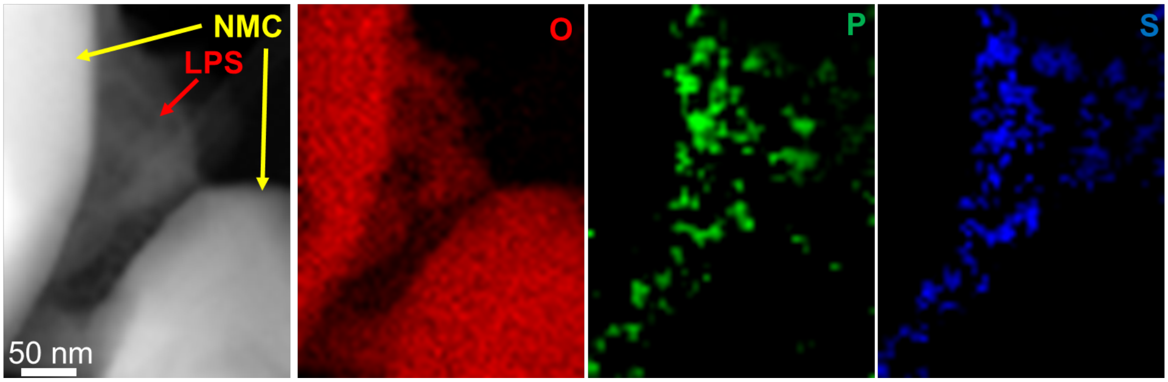

Figure S13. Composition characterization of cycled LBO-coated NMC^{532}. HADDF-STEM image and corresponding EELS maps of LBO-NMC and LPS composite after cycling from 2.5 to 4.3 V versus Li for 10 cycles.

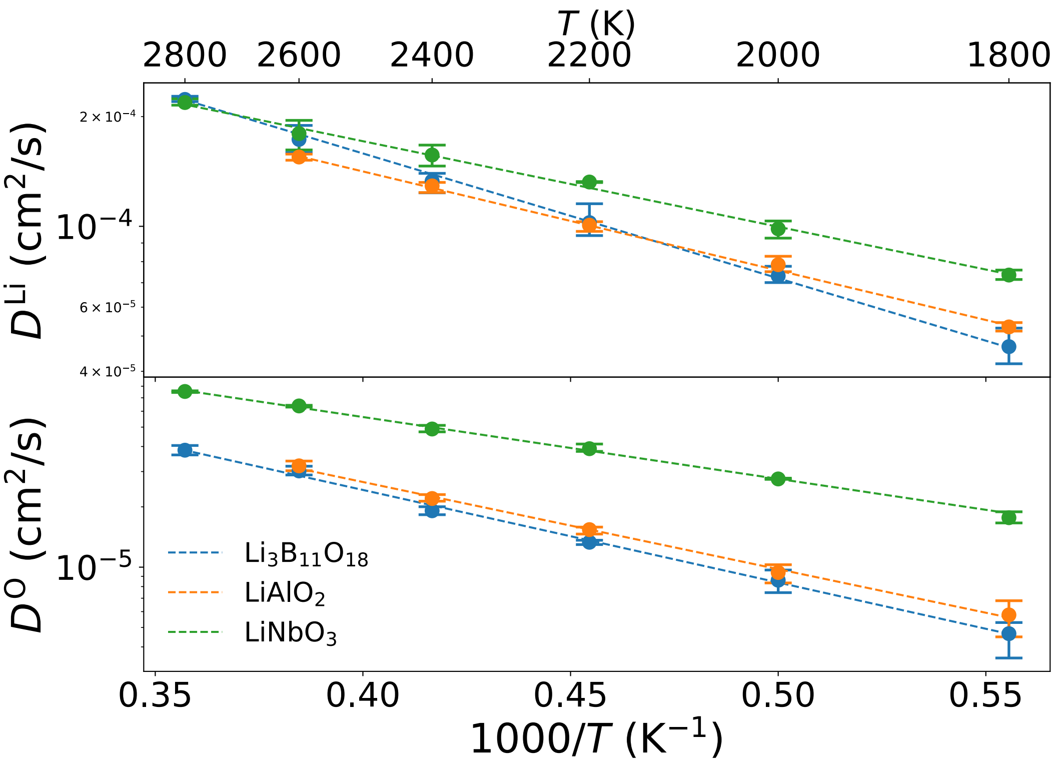

Figure S14. Arrhenius plots of Li^+ (a) and O^{2-} (b) diffusivity *D* as a function of temperature *T* in $Li_3B_{11}O_{18}$, $LiAlO_2$, and $LiNbO_3$. The error bar at each temperature represents the spread of *D* values as given by the three independent diffusion trajectories.

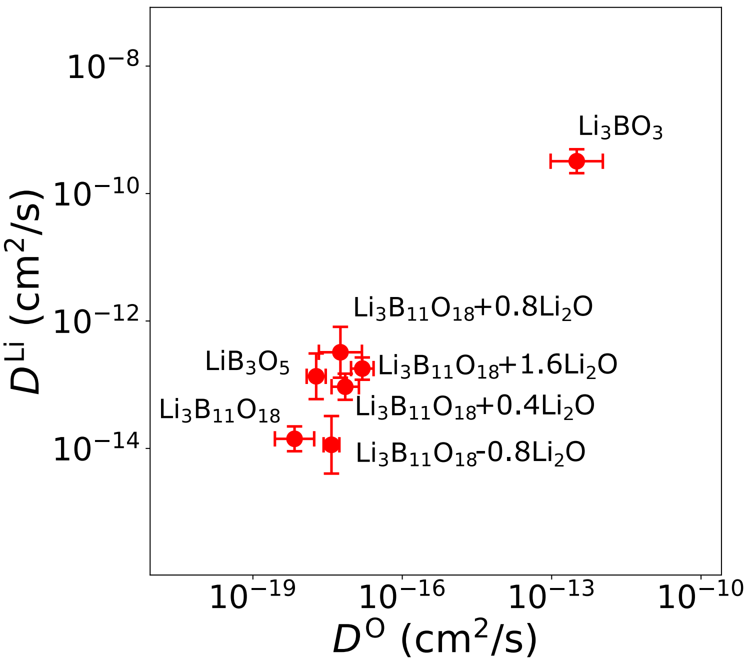

Figure S15. Calculated room-temperature diffusivities of Li^+ (D^{Li}) and O^{2-} (D^{O}) in Li-B-O compounds. The error bars represent the standard deviation of extrapolated self-diffusion coefficients at 300 K. $Li_3B_{11}O_{18}$ exhibits the smallest D^{O}.

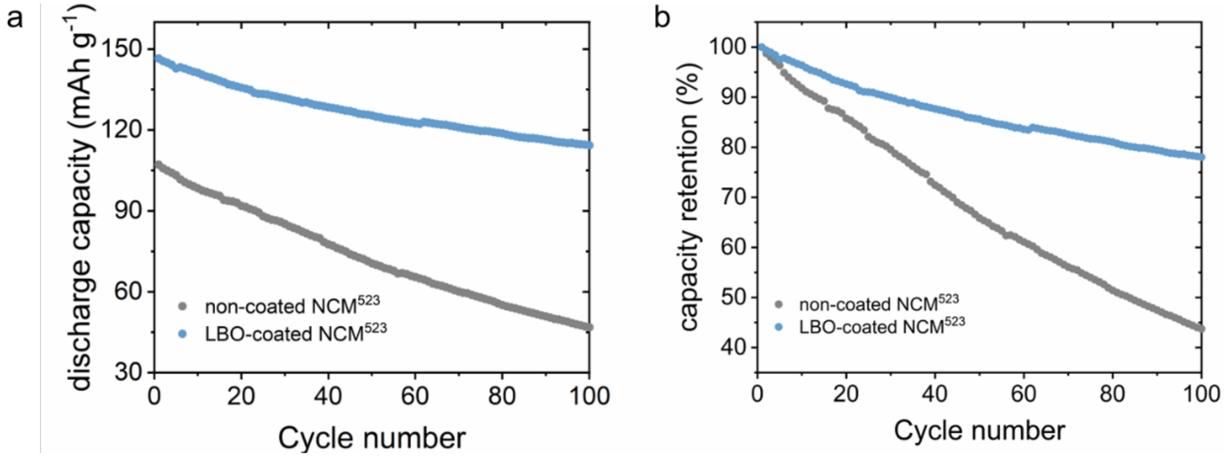

Figure S16. Cycling performance of non-coated and $Li_3B_{11}O_{18}$-coated $LiNi_{0.5}Co_{0.2}Mn_{0.3}O_2$. a. Discharge capacity and b. capacity retention of full cells using non-coated NCM and LBO-coated NCM as the cathode, LPS as the bulk SE, and graphite as the anode/reference electrode. The electrochemical data were obtained from reference[21] and are replotted here.

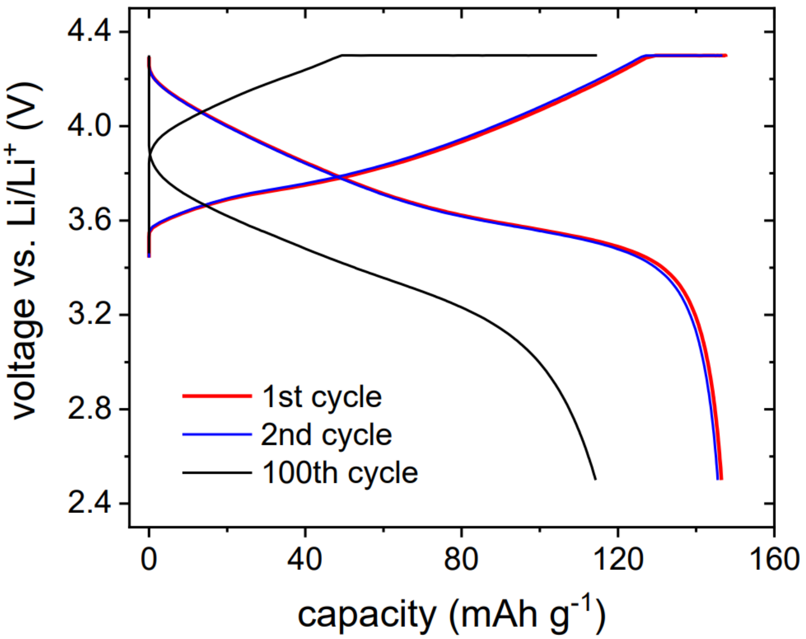

Figure S17. Electrochemical cycling of full cells using LBO-coated (red) NMC[532] cathodes. All the cells were subject to galvanostatic cycling (0.1 mA cm^{-2} for charge and 0.05 mA cm^{-2} for discharge) for 100 cycles. The electrochemical data were obtained from reference[21] and are replotted here.

Table S1. Estimated time t for O^{2-} to diffuse through the Al_2O_3 coating for various l_c and $\nabla\mu^i$. l_c is the Al_2O_3 coating thickness. μ_c^O and μ_e^O are the oxygen chemical potentials on the cathode and electrolyte side, respectively. We assume an NCM[523] cathode particle radius (r) of 5 µm that forms a surface layer of densified NiO rocksalt phase with thickness (l_s) of 2 nm. Amorphous 0.23 Li_2O·0.77 Al_2O_3 was used to simulate O^{2-} diffusion in Al_2O_3.

r (µm)	l_s (nm)	μ_c^O (eV)	μ_e^O (eV)	l_c (nm)	t (h)	Error bound t
5	2	-4.95	-8.39	1	2	0.6, 6.9
				10	19	6, 68
			-5.24	1	23	7.1, 81.5
				10	231	71, 812
